# Primary prevention of atrial fibrillation with beta-blockers in patients with end-stage renal disease undergoing dialysis

**DOI:** 10.1038/srep17731

**Published:** 2015-12-08

**Authors:** Ting-Tse Lin, Jiun-Yang Chiang, Min-Tsun Liao, Chia-Ti Tsai, Juey Jen Hwang, Fu-Tien Chiang, Jiunn-Lee Lin, Lian-Yu Lin

**Affiliations:** 1 Department of Internal Medicine, National Taiwan University Hospital Hsin-Chu Branch, Hsin-Chu, Taiwan; 2 Division of Cardiology, Department of Internal Medicine, National Taiwan University College of Medicine and Hospital, Taipei, Taiwan; 3 Department of Laboratory Medicine, National Taiwan University Hospital, Taipei, Taiwan

## Abstract

Current evidence suggests that beta-blocker lower the risk of development of atrial fibrillation (AF) and in-hospital stroke after cardiac surgery. This study was to assess whether beta-blockers could decrease incidence of new-onset AF in patients with end stage renal disease (ESRD). We identified patients from a nation-wide database called Registry for Catastrophic Illness, which encompassed almost 100% of the patients receiving dialysis therapy in Taiwan from 1995 to 2008. Propensity score matching and Cox’s proportional hazards regression model were used to estimate hazard ratios (HRs) for new-onset AF. Among 100066 patients, 41.7% received beta-blockers. After a median follow-up of 1500 days, the incidence of new-onset AF significantly decreased in patients treated with beta-blockers (HR = 0.483, 95% confidence interval = 0.437-0.534). The prevention of new-onset AF was significantly better in patients taking longer duration of beta-blockers therapy (P for time trend <0.001). The AF prevention effect remains robust in subgroup analyses. In conclusion, beta-blockers seem effective in the primary prevention of AF in ESRD patients. Hence, beta-blockers may be the target about upstream treatment of AF.

Atrial fibrillation (AF) is a result of continuous remodeling of the atrial, a dynamic interaction between a trigger and the substrate^1^,^2^. AF is increasingly associated with hypertension (HTN), congestive heart failure (CHF), diabetes mellitus (DM) and chronic kidney disease, all of which are recognized risk factors for the arrhythmia^3^,^4^,^5^. It is also prevalent after surgery, particular cardiothoracic surgery^6^. Postoperative AF (POAF) is likely related to pre-existing degenerative change in the atrial myocardium and perioperative conditions that result in abnormal electrophysiologic properties^7^,^8^. Potential adverse outcomes following postoperative AF include stroke, prolongation of hospital stay and death^9^,^10^.

Beta-blockers administration is the most widely used prophylactic strategy of POAF based on numerous studies showing benefit, ease of use and cost consideration^11^,^12^. The present guideline also recommends preoperative or early postoperative administration of beta-blockers in patients without contraindication in order to reduce the incidence of AF and clinical sequels after coronary bypass surgery^13^. This prophylactic therapy for POAF targets the sympathetic nervous system, atrial refractory period and conduction even though the mechanism of POAF is likely multifactorial^8^,^14^.

Patients with chronic kidney disease and end-stage renal disease (ESRD) are more likely to develop cardiovascular disease, including myocardial infarction^15^, sudden cardiac death^16^ and AF^17^,^18^. With regard to AF, poor controlled HTN and expansion of body fluid related to renal dysfunction lead to atrial stretch and fibrosis^19^. Pathological activation of renin-angiotensin-aldosterone system and systemic inflammation also create the required substrate for development of AF^20^,^21^. Although it is not clear whether patients with perioperative status and ESRD share common etiologies for AF, but both conditions have many similar factors prone to the development of AF.

In the AFFIRM study, beta-blockers were the most effectively and commonly used drug class for rate control^22^. Current guidelines recommend beta-blockers as one of rate control drugs, especially useful in the presence of high adrenergic tone or myocardium ischemia^23^,^24^. However, there is a paucity of studies concerning the role of beta-blockers on prophylactic effect for AF in ESRD patients. The present study was undertaken to assess the impact of treatment with beta-blockers on the development of AF in a large cohort of ESRD patients. We hypothesized that patients receiving beta-blockers would be associated with lower AF risks.

## Results

### Patient characteristics

**Figure 1:**
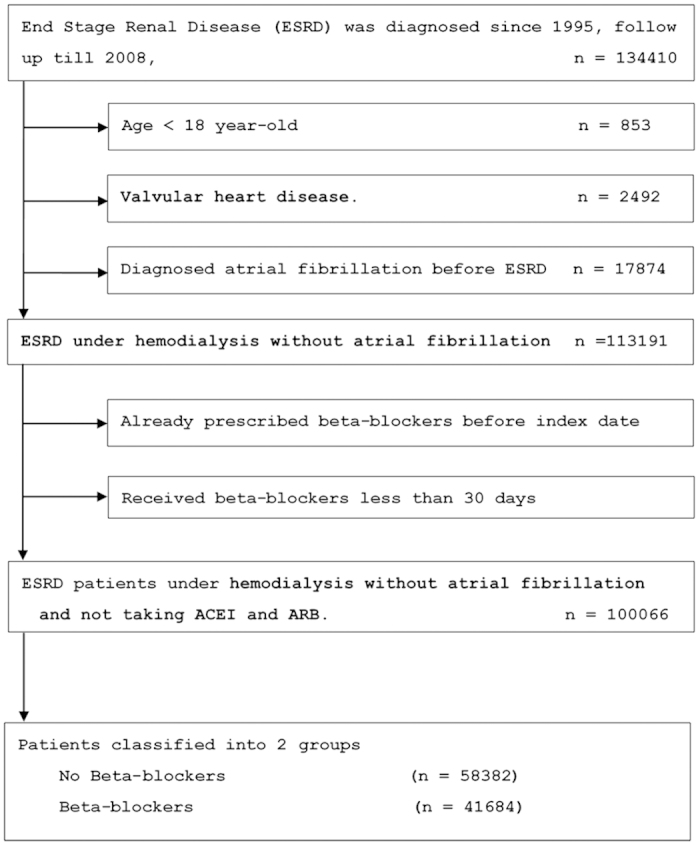
Patient flow diagram.

There were 100066 patients who met the study inclusion criteria; 58382 (58.3%) did not use beta-blockers while 41684 (41.7%) used beta-blockers. Patients not receiving beta-blocker treatment were served as control group. The median follow-up time was 1500 days. The algorithm was listed in Fig. 1.

Clinical and demographic characteristics were listed in Table 1. Patients without beta-blocker treatment were significantly elder than those with beta-blocker therapy and there were also significantly less female patients in non-beta-blocker group. The prevalence of receiving hemodialysis therapy was significantly higher in beta-blocker group (98.5%) than in control group (85.6%). As expected, the prevalence of risk factors including HTN (91.5% vs. 87.8%), DM (50.4% vs. 40.9%) and dyslipidemia (4.9% vs. 27.8%) were higher in beta-blocker group as well. The prevalence of comorbidities including ischaemic stroke/TIA (7.7% vs. 4.1%), hemorrhagic stroke (6.0% vs. 3.9%), CAD (50.2% vs. 29.2%), PAD (28.2% vs. 21.9%) and CHF hospitalization (27.2% vs. 21.3%) was also higher in beta-blocker group than in control group. Among the medication use, as compared with control group, ACEI/ARB (44.5% vs. 17.8%), calcium channel blockers (CCBs) (53.8% vs. 31.2%), diuretics (39.2% vs. 33.8%), statin (31.3% vs. 25.9%), OADs (29% vs. 23.3%) and insulin (19.2% vs. 7.3%) were more common in beta-blocker group. To minimize differences in the baseline characteristics between beta-blocker and non-beta-blocker group, patients were matched by using propensity method. As shown in Table 1, a total of 83340 patients were selected by propensity matching, the basic characteristics were matched well except in the use of ACEI/ARB and insulin.

### Main outcome: AF

The median durations of follow-up were 1295 and 1741 days in control and in beta-blocker group. As demonstrated in Table 2, the absolute incidence of new-onset AF during the entire follow-up period was less in beta-blockers group (1.5%) as compared with that in control group (4.9%) (Table 2). After transforming the incidence into patient-years, the incidence was still higher in control group (9.3 per 1000 patient-years) than in beta-blocker group (2.7 per 1000 patient-years).

**Figure 2:**
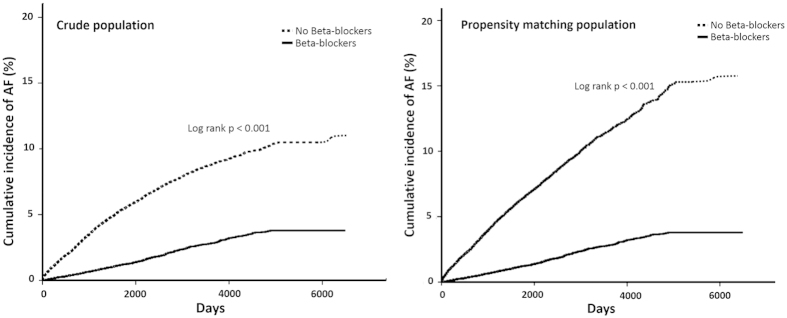
Kaplan–Meier curves showing the development of atrial fibrillation according to beta-blocker treatment.

The results of Cox’s regression analyses were demonstrated in Table 3. After adjusting for potential confounders, in comparison with the control group, use of beta-blockers (Model 1; adjusted HR, 0.483; 95% CI: 0.437-0.534) was associated with lower risk for developing AF. We observed similar results after propensity matching (Model 2; adjusted HR, 0.426; 95% CI: 0.347-0.512) and after further adjustment with propensity score (Model 3; adjusted HR, 0.488; 95% CI: 0.441-0.541)(Table 3). We stratified the treatment duration of beta-blockers into three categories including ≦60 days, 60-180 days and >180 days. For patients treated with beta-blockers, the adjusted HR were 0.559 (95% CI: 0.467-0.670), 0.472 (95% CI: 0.407-0.546) and 0.457 (95% CI: 0.398 to 0.524), respectively (Table 3). There was a dose-response relationship for duration of treatment and the prevention of new-onset AF (P < 0.05).The finding remained unchanged after propensity matching and adjustment. The Kaplan-Meier survival curves were illustrated in Fig. 2. We plotted survival curves based on crude population and matching population. The log-rank test was significant in beta-blocker vs. control group (P < 0.001).

**Figure 3:**
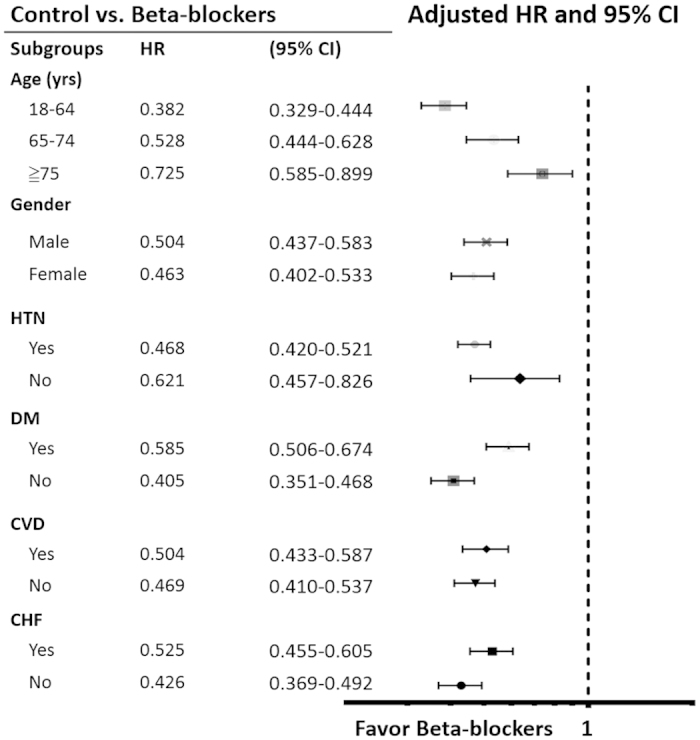
Relative risks of atrial fibrillation between patients with and without beta-blocker treatment, stratified by specific subgroups.

The results of subgroup analyses were demonstrated in Fig. 3. As shown in Fig. 3, the result that beta-blockers usage was associated with lower risk of new- onset AF remained unchanged in all subgroups (different age class, gender, HTN, DM, cardiovascular disease and CHF).

## Discussions

Currently, accepted primary prevention of AF was discouragingly limited. According to ESC 2012 guidelines, recent double-blind, placebo-controlled trials with ARBs and the majority of trials with polyunsaturated fatty acids failed to show convincing results^25^. Though some evidence existed for specific patient groups, such as ARB for heart failure patients^26^, patients with severe renal insufficiency were usually excluded from those studies^27^,^28^,^29^. In the meantime, patients with ESRD generally suffered from greater burden of AF, but primary prevention was less mentioned in the literature. The main finding from this nationwide database retrospective study of patients with ESRD is the protective effect of beta-blocker against development of AF, with an HR around 0.4 to 0.6 if the patient had received beta-blocker. A considerable percentage of the study population had other comorbidities, but consistent association between beta-blocker use and reduced incidence of AF was demonstrated from subgroup analysis. This might imply direct protective effect of beta-blocker in patient with ESRD, instead of simply treating the underlying comorbidities. The beneficial effect of beta-blocker was consistent irrespective of patients’ age, underlying comorbidities and even in patients treated with beta-blocker less than 2 months. Therefore, beta-blocker might potentially be an effective therapy for primary prevention in patients with ESRD.

### Impact of AF on ESRD

AF, while being the most common arrhythmia among general population, is also the most common arrhythmia among patients with ESRD^16^, accounting for 13% of patients on hemodialysis and 7% in patients undergoing peritoneal dialysis according to report in the large US Renal Data System (USRDS)^30^. Compared to general population, patients with ESRD suffer from even greater burden of AF, and with population aging, the numbers of patients with concomitant ESRD and AF will increase in the future^31^. The reason for more AF in patients with ESRD had been a topic receiving great attention in the past decades.

Both increased comorbid illnesses and non-traditional factors, such as inflammation and oxidative stress, contribute to the formation of substrate of AF in patients with ESRD^32^,^33^,^34^. Altered cardiac chambers dimensions and function were also in favor of AF development^35^. Compared to patients without AF, patients who developed AF had increased 4-year mortality and risk of stroke^36^. Contemporary guidelines has well-established recommendations regarding timing of anticoagulation, based on CHA_2_DS_2_VASc score, but currently there is insufficient evidence to support application of these recommendations to patients with ESRD. From prior study, warfarin was associated with inconsistent results in patients with ESRD, and was even a risk factor for stroke^37^. Furthermore, use of warfarin in patients with ESRD could double the risk of bleeding shown by two retrospective studies^38^,^39^. Given the clinical consequence, primary prevention of AF in patients with ESRD is of great importance.

### Primary prevention in AF

In the field of AF primary prevention, namely upstream therapy, effective therapy was discouragingly limited. ACEI/ARB did show its preventive effect in patients with HF with reduced LV ejection fraction from some retrospective studies^40^,^41^,^42^; however, this effect could not be reproduced in patients without structural heart disease in the Angiotensin II-antagonist in paroxysmal atrial fibrillation (ANTIPAF) trial^43^. In Japanese Rhythm Management Trial II for Atrial Fibrillation (J-RHYTHM II study), candesartan did not show its advantage over amlodipine in reducing AF frequency^44^. Statin was well known for the pleiotropic effect in cardiovascular disease, but meta-analysis showed that it could only reduce the risk of AF after coronary surgery^45^.

Several other pathophysiological processes, including sympathetic activation and oxidative stress, are associated with atrial remodeling that may predispose patients to AF, and could also be the targets of upstream therapy^46^. A representative example is beta-blockade in POAF. Given the condition with increased sympathetic tone and loss of vagal tone before POAF occurrence^47^ and the example of successful induction of AF by injecting isoproterenol and adrenaline into the sinus node artery in animal study^48^, beta-blockade reasonably became a therapeutic choice in preventing POAF. In a randomized control study, beta-blockade with short-term intravenous landiolol and long-term oral bisoprolol reduced up to 70% of POAF compared to control group^49^. To go a step further, biomarker tests demonstrated anti-ischemic, anti-inflammatory, and anti-oxidant effects brought by beta-blockade.

Patients with ERSD are also a special group in a milieu of high baseline sympathetic tone and oxidative stress^33^,^50^,^51^, and a retrospective study would serve well as the initial evaluation of potential benefit of beta-blockade. In addition to beta-blockade, prevent atrial substrate formation through RAAS-blockade is another therapeutic target since RAAS activation is also a frequent finding in patients with renal impairment^52^. Recently, our study group had found ACEI/ARB to be effective in reducing new-onset AF in patients with ESRD^53^, potentially indicating more contribution from RAS activation. This again emphasized on the unique pathophysiological characters and different therapeutic considerations of AF in patients with ESRD.

### Limitations

There are several limitations needed to be addressed. First, the study is limited by its retrospective, nonrandomized nature, and the imbalance in risk factors among different antihyperglycemic agents’ users in the whole cohort. Also, treatment allocation was not randomized and treatment selection bias had to be taken into account. Although we adjusted the confounding factors, the result might be still confounded by other underlying disease we did not consider. Second, our primary end-point was the time to the occurrence of AF diagnosed via ICD-CM coding. The accuracy of the diagnoses, which are based on the administrative data reported by physicians, may be a concern. Patients prescribed with beta-blocker might benefit more from beta-blocker originally, for example, more HF with reduced EF, which cannot be discriminated from patients with HF with preserved EF from the database. There is a risk that much AF is underreported because of diagnostic modality being used. Third, although we adjusted confounding factors, the result might be still confounded by other underlying disease we did not consider. Finally, we did not distinguish between chronic and paroxysmal AF and the results might differ in these two conditions.

## Conclusions

In patients with ESRD receiving replacement therapy, beta-blocker usage is associated with appeared to be effective in the primary prevention of AF.

## Materials and Methods

### Source of data

This large-scale, longitudinal cohort study used integrated medical and pharmacy claims data from National Health Insurance Research Database (NHIRD) in Taiwan. The National Health Insurance program has provided compulsory universal health insurance in Taiwan since 1995. More than 98% of the total Taiwanese population of 23 million is covered by the program. The NHIRD contains nearly complete claims history of diagnosis and procedures, provided as the International Classification of Diseases Ninth Revision Clinical Modification (ICD-9-CM) codes, and drug dispensing for every beneficiary. The NHIRD established a registry system for “Catastrophic Illnesses”, including cancer, chronic mental illness, congenital illness and ESRD. In Taiwan, ESRD patients undergoing hemodialysis and peritoneal dialysis are categorized as “severe illness” and the national insurance covers almost all the medical fees. All the medications, procedures, every out-patient clinic visits and hospital admission covered by the insurance were recorded in the database. The Bureau of National Health Insurance performs routine validations of the diagnoses by reviewing the original medical charts of all of the patients who applied for catastrophic illness registration. To comply with data privacy regulations, personal identities were encrypted and all data were analyzed in a de-identified manner

### Study population

We investigated the database of NHIRD during year of 1995 to 2008. The index date for the study cohort was identified as the date of the first-time that had both a diagnosis of ESRD (ICD9-CM: 585.6, 585.9, 586) and a procedure of hemodialysis (ICD9-CM: 39.93) or peritoneal dialysis (ICD9-CM: 54.98). These ESRD patients have already undergone dialysis treatment without receiving renal transplantation. We identified all patients who were above 18 years old. The exclusion criteria included the following: (1) diagnosis of valvular heart disease (ICD9-CM code: 394.X-396.X, 398.X), (2) prior ambulatory visit for AF (ICD9-CM code, 427.3), (3) receiving beta-blockers less than 30 days. A flowchart for the identification of study subjects is shown in Fig. 1.

### Drug use, covariates, and outcomes

Patients were classified into use and non-use of beta-blockers. We had 12 kinds of beta-blockers (around 90 of generic drugs with different doses). To simplify the presentation, we used the total treatment duration of beta-blockers to demonstrate the dose-response effect. The majority of the treatment frequencies for beta-blockers were once daily (qd; 20 to 30%) and twice a day (bid; 70 to 80%).

Comorbidity was defined by diagnoses at hospital discharge or in clinic records. For our study population, we searched the database to see if they had HTN(ICD-9-CM codes: 401.X-405.X), DM (250.X, 249.X), dyslipidemia (272.X), ischemic stroke (ICD9-CM code, 434.X), hemorrhagic stroke (ICD9-CM code, 430.X), CAD (coronary artery disease, ICD9-CM code, 411.X-414.X, V17.3, V81.0), CHF hospitalization (ICD9-CM code, 428.0-428.3, 429.9), PAD (peripheral artery disease, ICD9-CM code, 250.7, 443.X, 444.2). Medications that were dispensed at time of index date, including beta-blockers, angiotensin converting enzyme inhibitors (ACEI), angiotensin receptor blockers (ARB), calcium channel blockers (CCBs), diuretics, statin, oral anti-diabetic drugs (OADs) and insulin were identified.

The main purpose of the study was to compare long-term development of AF among patients with and without taking beta-blockers. Diagnosis of AF was based on ICD-9-CM coding (ICD9-CM code, 427.3) in any ambulatory visit and discharge diagnoses.

### Statistical analyses

For comparison of the baseline characteristics between two groups, Student’s t test was used for continuous variables and chi-square test was employed for categorical variables. Because patients were not randomly allocated to the two groups, we adjusted for age, gender, risk factors (HTN, DM and dyslipidemia), comorbidities (stroke/transient ischaemic accident, haemorrhagic stroke, CAD, PAD, CHF) and medication usage. In addition, the propensity score was applied to reduce the potential bias and to make the two groups more comparable. We derived a propensity score, which is the logit (probability) for receiving beta-blockers treatment from a logistic regression model by using all background covariates listed in Table 1 (age, gender, hemodialysis, HTN, DM, dyslipidemia, stroke, CAD, PAD, CHF hospitalization and medications). The predicted accuracy of the logistic model was assessed with an area under the receiver operating characteristic curve (C statistic), which was 0.864 (95% confidence interval [CI] 0.861–0.866). According to the propensity score, patients were selected by 1:1 matching without replacement using the nearest neighbor method. A caliper width of 0.15 standard deviations (SDs) was used for matching, because a width of between 0.05 and 0.30 SDs can eliminate almost 98% of the bias and correct the empirical type I error rates^54^. Baseline characteristics were compared within propensity score matched group.

For survival analyses, multivariate Cox’s proportional hazard regression analyses were used to derive the adjusted hazard ratios (HR) for developing AF by using patients without beta-blockers treatment as controls. Three models were used to adjust potential confounders. The model 1 was adjusted for age, gender, risk factors (HTN, DM and dyslipidemia), comorbidities (stroke/transient ischaemic accident, haemorrhagic stroke, CAD, PAD, CHF) and medication usage. The model 2 was similar to model 1 but included patients after propensity matching. In the model 3, the propensity score was entered as a covariate in the Cox’s proportional hazard regression model to adjust the potential selection bias. To test the consistency of the results, we also did subgroup analyses for different gender, age, presence of HTN, DM, cardiovascular disease (CVD) and CHF with adjustment for all confounders. The event-free survival curves of the two groups were illustrated by using the Kaplan-Meier method. The log-rank analysis was applied to test the differences in survival among groups.

All of the analyses were conducted using the Statistical Package for the Social Sciences (SPSS) for Windows, Version 19.0 (SPSS, Inc., Chicago, Illinois). A P value < 0.05 was considered statistically significant.

## Additional Information

**How to cite this article**: Lin, T.-T. *et al.* Primary prevention of atrial fibrillation with beta-blockers in patients with end-stage renal disease undergoing dialysis. *Sci. Rep.* **5**, 17731; doi: 10.1038/srep17731 (2015).

## Figures and Tables

**Table 1:** Patient baseline characteristics stratified by prescription of beta-blockers before and after propensity matching.

**Variables**	**Before matching**		**After matching**	
	**No BBs**	**With BBs**	**No BBs**	**With BBs**
N	58382	41684	41670	41670
Age (mean, yrs)	60	56*	56.7	56.2
Gender, female %	50.3	51.9*	51.0	51.9
Hemodialysis	82.5	98.5*	97.6	98.5
HTN, %	87.8	91.5*	89.6	91.5
DM, %	40.9	50.4*	48.6	50.4
Dyslipidemia	27.8	44.9*	41.2	44.9
Ischaemic stroke/TIA, %	4.1	7.7*	6.8	7.7
Haemorrhagic stroke, %	3.9	6.0*	5.6	6.0
CAD, %	29.2	50.2*	47.3	50.2
PAD, %	21.9	28.2*	25.6	28.2
CHF hospitalization, %	21.3	27.2*	25.4	27.2
Medications				
ACEI/ARB, %	17.8	44.5*	28.9	44.5*
CCBs, %	31.2	53.8*	46.9	53.8
Diuretics, %	33.8	39.2*	38.7	39.2
Statin, %	25.9	31.3*	28.9	31.3
OAD, %	23.3	29.0*	27.7	29.0
Insulin, %	7.3	19.2*	10.2	19.2*

*p < 0.05 compared with the no beta-blocker group.

ACEI, angiotensin converting enzyme inhibitor; AF, atrial fibrillation; ARB, angiotensin receptor blocker; BBs, beta-blockers; CAD, coronary artery disease; CCBs, calcium channel blocker; DM, diabetes mellitus; HTN, hypertension; HD, hemodialysis; CHF, congestive heart failure; TIA, transient ischaemic accident; OAD, oral anti-diabetic drugs; PAD, peripheral artery disease.

**Table 2:** Incidence of atrial fibrillation by prescriptions.

	**Incidence of AF**		
	**Total**	**No Beta group**	**Beta group**
Number of patients	100066	58382	41684
Duration of follow-up			
Median (IQR), days	1500 (551,3150)	1295 (333,3343)	1741 (807,2985)
Mean (SD), days	1955 (1643)	1911 (1773)	2017 (1439)
Incident cases - n (%)	3483 (3.4)	2860 (4.9)	624 (1.5)
Incidence per 1000 patient-years	6.5	9.3	2.7

Abbreviations: AF, atrial fibrillation; IQR, interquartile range; SD, standard deviation.

**Table 3:** Hazard ratios (95% CI) of developing atrial fibrillation in patients taking beta-blockers, with no beta-blocker treatment as the reference group.

	Model 1*		**Model 2**†		**Model 3**††	
	**HR**	**95% CI**	**HR**	**95% CI**	**HR**	**95% CI**
Overall						
AF incidence	0.471	0.426–0.521	0.483	0.437–0.534	0.488	0.441–0.541
Period of treatment ≦60 days						
AF incidence	0.559	0.467–0.670	0.631	0.526–0.757	0.561	0.468–0.672
Period of treatment 60 to180 days						
AF incidence	0.472	0.407–0.546	0.518	0.446–0.601	0.475	0.410–0.550
Period of treatment >180 days						
AF incidence	0.457	0.398–0.524	0.513	0.445–0.591	0.465	0.405–0.534
P for time trend	<0.001	<0.001	<0.001			

The Hazard ratios were also stratified by different period (number of days) of beta-blockers treatment, with no beta-blocker treatment as the reference group. AF, atrial fibrillation; ACEIs, angiotensin converting enzyme inhibitors; ARB, angiotensin receptor blockers; CCBs, calcium channel blockers, HR, hazard ratio; OADs, oral anti-diabetic drugs.

^*^Model 1: adjusted for age, gender, risk factors (hypertension, diabetes mellitus and dyslipidemia), comorbidities (stroke/transient ischaemic accident, haemorrhagic stroke, coronary artery disease, peripheral artery disease, heart failure), and medication usage (ACEI/ARB, CCBs, diuretics, OADs, insulin)

^†^Model 2: adjusted all variables in Model 1 after propensity score matching

^††^Model 3: adjusted all variables in Model 1 plus propensity score adjustment.
